# Psychological resilience and marital adjustment in breast cancer patients: a multi-center study of serial mediation by anxiety and depression

**DOI:** 10.3389/fpsyg.2026.1789074

**Published:** 2026-05-29

**Authors:** Yanting Ning, Liqiong Liu, Jianying Kuang, Yuqun Wei, Nan Shen, Luhong Yang, Wenqing Wu, Yang Yu, Wennian Cai, Siqin Li

**Affiliations:** National Cancer Center/National Clinical Research Center for Cancer/Cancer Hospital and Shenzhen Hospital, Chinese Academy of Medical Sciences and Peking Union Medical College, Shenzhen, China

**Keywords:** anxiety, breast neoplasms, depression, marital adjustment, psychological resilience

## Abstract

**Background:**

Breast cancer patients experience significant psychological distress and marital strain during treatment. Psychological resilience may protect marital adjustment through emotional pathways, yet evidence of serial mediation mechanisms remains limited in nursing research.

**Objective:**

To examine anxiety and depression as serial mediators between psychological resilience and marital adjustment.

**Methods:**

A multicenter cross-sectional study recruited 408 breast cancer patients from oncology departments in Shenzhen, Beijing, and Hebei. Standardized instruments assessed psychological resilience (Connor-Davidson Resilience Scale, CD-RISC), anxiety/depression (Hospital Anxiety and Depression Scale, HADS), and marital adjustment (Dyadic Adjustment Scale, DAS). Serial mediation analysis (PROCESS Macro Model 6) with 5,000 bootstrap resamples was conducted, adjusting for 15 clinical and sociodemographic covariates.

**Results:**

Psychological resilience significantly correlated with marital adjustment (*r* = 0.295, *p* < 0.001). In the adjusted model, the total indirect effect was significant (*b* = 0.514, 95% CI [0.211, 0.839]), accounting for 53.65% of the total effect. The serial indirect pathway (resilience → anxiety → depression → marital adjustment) was significant (*b* = 0.219, 95% CI [0.069, 0.403]), explaining 22.86% of the total effect. The simple indirect pathway through depression was also significant (*b* = 0.153, 95% CI [0.047, 0.292]), while the pathway through anxiety alone was not. The direct effect remained significant (*b* = 0.444, 95% CI [0.076, 0.812]).

**Conclusion:**

Psychological resilience is positively associated with marital adjustment through sequential associations with lower anxiety and depression. Oncology nurses should implement resilience-focused psychoeducation and couples’ communication training, particularly targeting anxiety in the early treatment phase, to enhance patients’ perceived marital adjustment.

## 1 Background

Breast cancer remains one of the most prevalent malignancies worldwide, affecting over 2.3 million women annually and imposing profound psychological and relational burdens on patients and their families (Giaquinto et al., 2024). Beyond the physical toll of diagnosis and treatment, survivors often grapple with heightened emotional distress (Luz Patricia et al., 2025). Meta-analytic studies reveal that 34 to 41.9% of women with breast cancer experience significant anxiety during treatment (Fortin et al., 2021; Hashemi et al., 2020), while 20 to 32.2%face major depressive episodes (Fortin et al., 2021, Pilevarzadeh et al., 2019). Furthermore, a series of problems faced by breast cancer patients, such as encompassing fertility loss, treatment side effects, and ongoing psychological distress, have heightened their marital discord (Tarawneh et al., 2024; Caruso et al., 2024). It was reported that the prevalence of sexual dysfunction ranged from 17.5% before BC diagnosis to 86% after 6 months of hormone therapy (Rodrigues-Machado et al., 2025). The rate of divorce among breast cancer survivors is higher compared to the general population (Dorval et al., 1999), and marital distress is an independent predictor of decrease in 10-year survival rates (Coyne, 2010). In such circumstances, marital adjustment, defined as the couple’s collective ability to manage stress related to illness (Chen et al., 2024), becomes a significant factor affecting prognosis rather than just a matter of quality of life.

Psychological resilience is recognized as a changeable asset that mitigates this decline by promoting emotional stability and adaptable coping strategies (Mohlin et al., 2021; Mehrabizadeh et al., 2024), but it is unclear how this may be achieved within the acute context. There is developing evidence supporting that resilience can work as a modifiable protective factor for onset and persistence of emotional dysfunction, with recent studies extending from correlational analyses to mechanistic inquiries (Li et al., 2020; Ding et al., 2025) and therapeutic booster interventions (Zhang et al., 2025). Resilient women can be pragmatic about managing cancer related stress, and consequently have more moderate emotional reactions. The association of depression and resilience to marital adjustment is explored in previous studies which implied that (i) psychological resilience can improve the level of marital satisfaction, (ii) more depression will be associated with less quality on the spousal relationships in infertile couples (Bhamani et al., 2022).

The explanation of the changes in these marital relationships is posited through Karney and Bradbury’s (1995) Vulnerability-Stress-Adaptation (VSA) model, which suggests that the quality of a marriage is shaped by concurrent levels of inherent vulnerabilities, existing stressors, and adaptive processes within the relationship. Breast cancer, as a psychosocial stressor that heightens anxiety and depression, creates emotional vulnerabilities. Partners may mitigate these by influencing the patient’s ability to control her feelings and offer support (Weber et al., 2023). Psychological resilience could, therefore, be considered as an adaptive reserve that assists the individual to better regulate interpersonal weaknesses (Lai et al., 2020).

Similarly, while breast cancer patients undergo unique life-course changes and social role stressors that may differentially impact relational stress processes as compared to women with other cancers, it is a question yet to be examined. Stress Coping Theory (Lazarus, 1984) provides a framework to understand how individuals appraise and cope with stress (Lazarus, 1984; Folkman et al., 1986a, 1986b. People first consider the threat, and then they focus on coping resources. Nonetheless, the increasing total of strains following treatment may initiate depressive symptoms (Chen et al., 2025). These patterns point to a sequential emotional process whereby resilience may shape marital outcomes through its influence on anxiety and subsequent depressive symptoms.

While the relationship between anxiety, depression, and marital adjustment in breast cancer populations has garnered attention, there are still gaps in the existing research (Lau et al., 2025). Studies have shown that anxiety typically peaks post-diagnosis, whereas depression tends to emerge later (Lamers et al., 2011; Kalin, 2020), suggesting a temporal sequence in their manifestation. However, the potential role of anxiety and depression as sequential mediators in the connection between psychological resilience and marital adjustment remains inadequately explored (Zou et al., 2024). Existing studies often treat anxiety and depression independently as mediators between resilience and marital functioning, overlooking their potential sequential interplay (Eide et al., 2025; Kong et al., 2024; Li et al., 2015). Consequently, the specific mechanisms through which resilience influences emotional distress associated with breast cancer and subsequently affects marital well-being remain unclear.

To strengthen the theoretical basis for the serial pathway, we integrate the Vulnerability-Stress-Adaptation (VSA) model and Stress-Coping Theory (SCT). The VSA model positions psychological resilience as a modifiable protective factor that reduces vulnerability to cancer stressors. These stressors first elicit anxiety, which then contributes to depression; the resulting emotional distress impairs dyadic coping and marital adjustment. This mechanism is particularly relevant for breast cancer patients facing unique relational challenges (Cihan and Aydogan, 2020).

SCT explains the temporal ordering of anxiety preceding depression through sequential cognitive appraisals, with resilience facilitating adaptive coping that prevents escalation (Li et al., 2022). SCT and the VSA model are complementary: SCT accounts for individual-level emotional processes, while VSA explains how these processes disrupt couple-level adaptation (Ross et al., 2022). Together they provide a clear rationale for the resilience-anxiety-depression-marital adjustment pathway and support targeted interventions.

Recent meta-analyses have demonstrated that couple-based psychosocial interventions significantly improve marital adjustment, dyadic coping, and psychological outcomes in breast cancer dyads (Badr and Krebs, 2013; Bidstrup et al., 2015). These findings underscore the clinical importance of targeting relational processes. Longitudinal studies further establish that anxiety symptoms typically emerge earlier and at higher intensity than depressive symptoms in the first year post-diagnosis (Regan et al., 2012), consistent with cognitive appraisal models in which anxiety represents the initial response to threat, while depression develops under sustained unmitigated stress. Although the reverse pathway (depression → anxiety) has been observed in some chronic conditions, empirical trajectory data in breast cancer populations and theoretical considerations of acute illness-related threat appraisal support anxiety as the proximal mediator in the present model.

In the present research, we constructed and assessed a serial-mediation model where psychological resilience impacts marital adjustment by sequentially influencing anxiety and depression. Drawing upon the VSA model and SCT, and in light of the temporal progression of emotional distress, this study aims to clarify basic psychological links related between individuals’ coping capacities with partnership functioning during cancer. The study hypothesized: (1) that increased psychological resilience would be positively associated with marital adjustment; (2) anxiety and depression would mediate the relationship between psychological resilience and marital adjustment; and, (3) one potential indirect cascade from high levels of psychological resilience through decreased anxiety to decreased depression resulting in higher levels of marital adaptation. Results are expected to inform the development of theoretically-driven, personalized psychosocial interventions for breast cancer patients and their supporters.

## 2 Methods

### 2.1 Study design and participants

This study was a quantitative, cross-sectional design. We used the Strengthening the Reporting of Observational Studies in Epidemiology (STROBE) statement guidelines to report this study (Supplementary Table S1) (von Elm et al., 2007). This multicenter cross-sectional study recruited 408 breast cancer patients from oncology inpatient wards at tertiary hospitals in Shenzhen, Beijing, Hebei. A pilot survey was conducted in December 2024 (*n* = 30) to pretest and refine the questionnaire for clarity and cultural appropriateness. The main data collection took place between April and June 2025 using convenience sampling. A total of 452 eligible breast cancer patients were consecutively approached during the study period. Of these, 408 patients provided informed consent and completed the questionnaires, yielding a response rate of 90.3%. The main reasons for non-participation among the 44 patients who declined were: lack of time or interest (*n* = 28), feeling too unwell to complete the questionnaire (*n* = 11), and concerns about privacy (*n* = 5). Due to the real-world clinical setting and time constraints of inpatient care, probability sampling was not feasible; therefore, convenience sampling was employed. While this approach allowed efficient recruitment of a relatively large sample within the study timeframe, it may limit generalizability to the broader breast cancer population, particularly patients treated in non-tertiary hospitals or those with more advanced disease. We have therefore added a dedicated statement on this limitation in the Discussion section. Eligible participants were women aged 20–83 years with histologically confirmed stage I-III breast cancer, married or cohabiting for at least 1 year, and without cognitive impairment or severe psychiatric comorbidities.

### 2.2 Data collection procedures

A structured questionnaire was designed to capture essential variables, including sociodemographic [age, ethnicity, educational level, occupation, and monthly income categories (Table 1)], and clinical characteristics (marital duration and number of children; fertility intention; time since diagnosis; surgery type [breast-conserving surgery, mastectomy, or reconstruction]; health insurance status; sexual frequency changes after treatment [decreased, stable, or increased], pain severity evaluated by an 11-point Numeric Rating Scale-NRS (0 = no pain, 10 = worst imaginable pain). Cancer stage, chemotherapy/radiotherapy/endocrine therapy status, and menopausal status were not collected in the final questionnaire. In our preliminary pilot survey, we attempted to obtain self-reported cancer stage; however, a large proportion of patients either did not know their stage or provided inaccurate information. To avoid measurement error and minimize patient burden during active treatment, these variables were excluded from the main study. Prior to questionnaire administration, the researchers informed participants about the purpose and content of the study. Participants were given enough time to fill the questionnaire. If participants did have any problems, or questions during their participation in the exercise, trained researchers were on hand to assist.

**Table 1:** Characteristics of the participants (*n* = 408).

Variables	Total (*n* = 408)
Anxiety, *M* (Q_1_, Q_3_)	7.000 (4.000, 9.000)
Depression, *M* (Q_1_, Q_3_)	5.000 (2.000, 9.000)
Resilience, *M* (Q_1_, Q_3_)	27.000 (21.000, 32.000)
Marital adjustment, *M* (Q_1_, Q_3_)	111.000 (89.250, 127.000)
Pain scores, *M* (Q_1_, Q_3_)	1.500 (0.000, 3.000)
Age, *n* (%)	
20–30 years	16 (3.922)
30–40 years	112 (27.451)
40–50 years	181 (44.363)
>50 years	99 (24.265)
City, *n* (%)	
Shenzhen	262 (64.216)
Hebei	32 (7.843)
Beijing	104 (25.490)
Other cities	10 (2.451)
Ethnicity, *n* (%)	
Han	382 (93.627)
Ethnic minority	26 (6.373)
Education, *n* (%)	
Junior high school and below	102 (25.000)
High school/vocational school	105 (25.735)
College associate	79 (19.363)
Bachelor’s degree or above	122 (29.902)
Occupation, *n* (%)	
Employed	229 (56.127)
Retirement	58 (14.216)
Unemployed (including farming)	121 (29.657)
Income monthly (CNY), *n* (%)	
<2,000 CNY	61 (14.951)
2,001–5,000 CNY	131 (32.108)
5,001–10,000 CNY	115 (28.186)
>10,000 CNY	101 (24.755)
Marital status, *n* (%)	
First marriage	394 (96.569)
Remarried or above	14 (3.431)
Marriage duration, *n* (%)	
1–10 years	76 (18.627)
11–20 years	146 (35.784)
21–30 years	103 (25.245)
>30 years	83 (20.343)
Children status, *n* (%)	
Yes	382 (93.627)
No	26 (6.373)
Fertility intention, *n* (%)	
Yes	39 (9.559)
No	335 (82.108)
Not sure	34 (8.333)
Years of diagnosis, *n* (%)	
<1 year	282 (69.118)
1-2 years	83 (20.343)
3–5 years	17 (4.167)
>5 years	26 (6.373)
Insurance type, *n* (%)	
Public funds	5 (1.225)
Medical insurance	388 (95.098)
Self-funded	15 (3.676)
Surgical type, *n* (%)	
Breast-conserving surgery	194 (47.549)
Mastectomy	193 (47.304)
Breast reconstruction	21 (5.147)
Sexual frequency, *n* (%)	
No significant change	157 (38.480)
Decreased frequency	249 (61.029)
Increased frequency	2 (0.490)
Anxiety (HADS-A), *n* (%)	
<8	241 (59.069)
≥8	167 (40.931)
Depression (HADS-D), *n* (%)	
<8	270 (66.176)
≥8	138 (33.824)

Continuous variables presented as median (Q1, Q3) due to non-normal distribution (Shapiro–Wilk *p* < 0.001).

### 2.3 Sample size justification

Sample size determination was carried out with Monte Carlo simulation-based power analysis specific to serial multiple mediation models as initially outlined by Fritz and Mackinnon (2007) and later extended by Schoemann et al. (2017) With a small to moderate indirect effect size (*a* * *b* = 0.09–0.12), an 80% power, *α* = 0.05 (two-tailed), and 5,000 replications. Path coefficients were specified to reflect the assumed indirect effect magnitude, and bias-corrected bootstrap confidence intervals were used to evaluate statistical significance. The simulation showed we would need at least *n* = 350 subjects model in order to detect this serial mediated effect with appropriate precision for confidence interval (Preacher and Hayes, 2008; Muthén and Muthén, 2002). To ensure sufficient power for multivariable adjustment (15 covariates) and to account for potential missing or ineligible data, we oversampled beyond this minimum requirement. The final analytic sample (*N* = 408) exceeded the estimated threshold, supporting the adequacy of statistical power for the planned analyses (Hayes, 2022).

### 2.4 Measures

#### 2.4.1 Hospital Anxiety and Depression Scale (HADS)

The Hospital Anxiety and Depression Scale is a frequently employed 14-item self-report tool for assessing anxiety and depression in general hospital patients. Unlike the PHQ-9 and GAD-7, which include somatic symptoms that may overlap with physical illness, the HADS deliberately excludes somatic items, making it particularly suitable for cancer populations where physical symptoms are prevalent. This advantage has been widely recognized in psycho-oncology research. This instrument, comprising two subscales, namely HADS-A for anxiety (7 items) and HADS-D for depression (7 items), demonstrates robust psychometric properties, particularly within cancer populations (Norton et al., 2013). Responses are rated on a 4-point Likert scale, with scores equal to or exceeding 8 on either subscale indicative of clinically significant symptoms (Zigmond and Snaith, 1983). In the present study, the Cronbach’s *α* for the total scale was 0.899. The Cronbach’s *α* were 0.855 for the HADS-A subscale and 0.821 for the HADS-D subscale.

#### 2.4.2 Connor-Davidson Resilience Scale, 10-item version (CD-RISC-10)

The CD-RISC-10 is a concise and dependable tool utilized for assessing an individual’s resilience in five key domains: flexibility, self-efficacy, emotion regulation, optimism, and attention focus (Campbell-Sills and Stein, 2007; Connor and Davidson, 2003). This scale comprises 10 items, each rated on a 5-point Likert scale ranging from 0 (“not true at all”) to 4 (“true nearly all the time”), resulting in a total score range of 0 to 40. Higher scores on this scale are indicative of higher levels of resilience. Previous studies have demonstrated satisfactory internal consistency of this scale (Cheng et al., 2020). In the present study, a Cronbach’s alpha coefficient of 0.949 was obtained, further affirming its reliability.

#### 2.4.3 Dyadic Adjustment Scale (DAS-32)

The Dyadic Adjustment Scale is a commonly employed tool to evaluate marital adjustment and relationship satisfaction within dyadic relationships (Spanier, 1976; Chang et al., 2004). Comprising 32 items distributed among four domains-dyadic consensus, satisfaction, cohesion, and emotional expression. The total scores between 0 and 151, with higher scores reflecting increased relationship satisfaction. The original English version was developed by Spanier (1976). The Chinese version of the DAS has been validated and widely used in Chinese populations, demonstrating satisfactory reliability and validity (Shek, 1995). It should be noted that the DAS was administered solely to patients; therefore, the scores reflect patients’ subjective perceptions of their marital relationship rather than objective dyadic functioning. The Cronbach’s *α* coefficient for the overall scale in this investigation was calculated at 0.954.

### 2.5 Statistical analysis

All data analyses were performed using IBM SPSS Statistics (version 26.0) and Hayes (2022) PROCESS macro (version 3.4.1). A fixed random seed (12345) was used to ensure the reproducibility of bootstrap procedures and multiple imputation. Categorical variables were presented as frequencies and percentages. The non-normal distribution of continuous variables was confirmed through Shapiro–Wilk tests (*p* < 0.001), leading to the reporting of medians and interquartile ranges (IQRs). Although the distributions of continuous variables deviated from normality (Shapiro–Wilk *p* < 0.001), linear regression-based models were retained for mediation analysis. This decision was based on the robustness of ordinary least squares (OLS) estimation to non-normality in large samples. In the present study, bias-corrected bootstrap confidence intervals (5,000 resamples) were used to estimate indirect effects, which do not rely on normality assumptions (Alfons et al., 2022). Partial correlations were used to examine the relationships among resilience, anxiety, depression, and marital adjustment, while adjusting for 15 sociodemographic and clinical covariates. Missing data (<2%) were handled using multiple imputation by chained equations (MICE), generating 5 imputed datasets under the assumption of missing at random.

Mediation analyses were conducted separately within each imputed dataset using PROCESS Model 6. Parameter estimates were then pooled across imputations according to Rubin’s (1987) rules to obtain final coefficients and standard errors. Multicollinearity was assessed using variance inflation factors (VIF) derived from regression models in which depression was specified as the dependent variable and anxiety, resilience, and all covariates were entered as independent variables. VIF values <5 were considered indicative of no significant multicollinearity. Sensitivity analyses showed comparable results between imputed and complete-case datasets (<5% difference). Serial mediation analysis was conducted using PROCESS Model 6 to evaluate the indirect pathway from resilience to marital adjustment via anxiety and depression. All covariates were included in the adjusted model. Indirect effects were estimated using 5,000 bias-corrected bootstrap samples, with statistical significance denoted by 95% confidence intervals that did not include zero. Effect sizes were presented as unstandardized coefficients and proportions of indirect/total effects. Model fit was evaluated using *R*^2^. The robustness of the findings was evaluated by comparing adjusted and unadjusted models through absolute percentage change (|Δ|) and confidence interval overlap, following predefined thresholds (Vanderweele, 2015).

### 2.6 Ethical considerations

The study received approval from the Institutional Review Board of the National Cancer Center/National Clinical Research Center for Cancer/Cancer Hospital & Shenzhen Hospital, Chinese Academy of Medical Sciences and Peking Union Medical College (Approval No. KYKT2025-3-1) in compliance with the Declaration of Helsinki. All participants provided written informed consent and the participants could withdraw anytime without treatment compromise.

## 3 Results

### 3.1 Characteristics of the study participants

408 participants were included in the study, with the predominant age group being 40–50 years old (44.363%). The majority were ethnic Han (93.627%), and a significant number resided in Shenzhen (64.216%). 69.118% had a disease duration of under 1 year. The primary surgical procedures were breast-conserving surgery (47.549%) and mastectomy (47.304%), as indicated in Table 1. The median anxiety score was 7.0 (IQR: 4.0–9.0), with 40.931% of participants scoring ≥8 on the HADS-A (clinical cutoff). The median depression score was 5.0 (IQR: 2.0–9.0), with 33.824% of participants scoring ≥8 on the HADS-D. These rates are generally consistent with or slightly higher than those reported in recent Chinese breast cancer studies (anxiety: 34–42%; depression: 20–32%) (Pilevarzadeh et al., 2019; Hashemi et al., 2020).

### 3.2 Relationships among the variables

After adjusting for all covariates, marital adjustment among breast cancer patients remained significantly positively correlated with resilience (*r* = 0.295, *p* < 0.001), and significantly negatively correlated with both anxiety (*r* = −0.307, *p* < 0.001) and depression (*r* = −0.345, *p* < 0.001). Additionally, resilience remained significantly negatively associated with anxiety (*r* = −0.532, *p* < 0.001) and depression (*r* = −0.535, *p* < 0.001), while a significant positive correlation between anxiety and depression was also observed (*r* = 0.709, *p* < 0.001) (Table 2).

**Table 2:** Correlations among depression, anxiety, resilience, and marital adjustment.

Variable	1	2	3
1. Marital adjustment (DAS)	1		
2. Resilience (CD-RISC)	0.295***	1	
3. Anxiety (HADS-A)	−0.307***	−0.532***	1
4. Depression (HADS-D)	−0.345***	−0.535***	0.709***

DAS, Dyadic Adjustment Scale (marital adjustment); CD-RISC, Connor-Davidson Resilience Scale (resilience); HADS-A, Hospital Anxiety and Depression Scale-Anxiety Subscale (anxiety); HADS-D, Hospital Anxiety and Depression Scale-Depression Subscale (depression); ***p < 0.001; Correlations were adjusted for the following covariates: pain, income, age, education, sexual activity, surgery type, insurance type, time since diagnosis, fertility intention, ethnicity, occupation, marital status, marriage duration, children status, and city.

### 3.3 Serial mediation analysis

In the regression model with depression as the dependent variable and anxiety, resilience, and covariates as predictors, VIF values ranged from 1.045 to 2.023, indicating no evidence of multicollinearity. Specifically, no problematic collinearity was observed between anxiety and depression.

**Figure 1:**
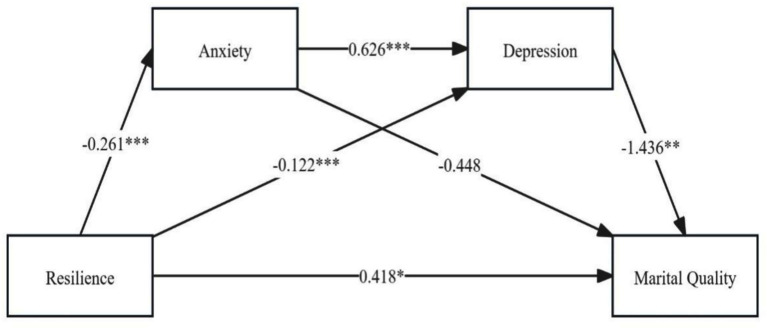
Serial mediation model (crude model). ***p < 0.001; **p < 0.01; *p < 0.05.

In the crude model, the total indirect effect was significant (*b* = 0.526, SE = 0.165, 95% CI [0.216, 0.866]), accounting for 55.72% of the total effect. Specific indirect effect 1 (resilience → anxiety → marital adjustment) was not significant (*b* = 0.117, SE = 0.145, 95% CI [−0.162, 0.405]). Specific indirect effect 2 (resilience → depression → marital adjustment) was significant (*b* = 0.174, SE = 0.070, 95% CI [0.058, 0.329]), accounting for 18.54% of the total effect. Specific indirect effect 3 (resilience → anxiety → depression → marital adjustment) was significant (*b* = 0.235, SE = 0.089, 95% CI [0.080, 0.424]), accounting for 24.89% of the total effect (Table 3; Figure 1).

**Table 3:** Chain mediation effects of resilience on marital adjustment via anxiety-depression pathway.

Effect type	Path	Crude model				Adjusted model			
		Coeff (*b*)	SE	95% CI	Effect proportion	Coeff (*b*)	SE	95% CI	Effect proportion
Direct effect	*X* → *Y*	0.418	0.187	[0.050, 0.785]	44.28%	0.444	0.187	[0.076, 0.812]	46.35%
Effect 1	*X* → M1 → *Y*	0.117	0.145	[−0.162, 0.405]	12.39%	0.142	0.15	[−0.141, 0.448]	14.82%
Effect 2	*X* → M2 → *Y*	0.174	0.070	[0.058, 0.329]	18.54%	0.153	0.063	[0.047, 0.292]	15.97%
Effect 3	*X* → M1 → M2 → *Y*	0.235	0.089	[0.080, 0.424]	24.89%	0.219	0.085	[0.069, 0.403]	22.86%
Total indirect effect		0.526	0.165	[0.216, 0.866]	55.72%	0.514	0.163	[0.211, 0.839]	53.65%
Total effect		0.944	0.156	[0.638, 1.249]	100.00%	0.958	0.157	[0.6492, 1.266]	100.00%
*R* ^2^		0.083				0.156			

*X*, resilience; M1, anxiety; M2, Depression, *Y*, marital adjustment; Adjusted model: controlled for 15 covariates: age, education, income, ethnicity, occupation, marital status, marriage duration, children, residence, pain score, sexual activity, surgery type, insurance, time since diagnosis, and fertility intention; Brackets contain 95% bias-corrected bootstrap CIs (5,000 resamples).

**Figure 2:**
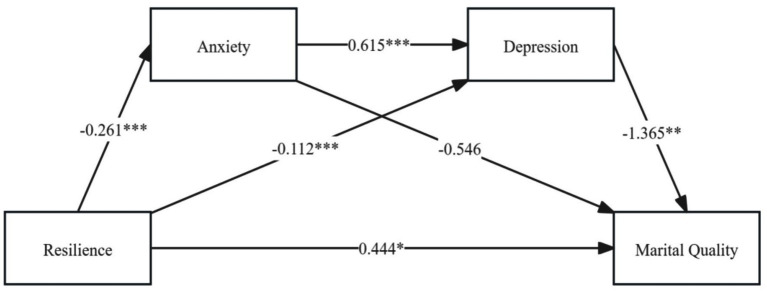
Serial mediation model (adjusted model). ***p < 0.001; **p < 0.01; *p < 0.05.

In the adjusted model, the total indirect effect was significant (*b* = 0.514, SE = 0.163, 95% CI [0.211, 0.839]), accounting for 53.65% of the total effect. Specific indirect effects 2 (*b* = 0.153, SE = 0.063, 95% CI [0.047, 0.292]) and 3 (*b* = 0.219, SE = 0.085, 95% CI [0.069, 0.403]) were significant, contributing 15.97 and 22.86% to the total effect, respectively. Specific indirect effect 1 remained non-significant (*b* = 0.142, SE = 0.150, 95% CI [−0.141, 0.448]). The model explained 15.6% of the variance in marital adjustment (*R*^2^ = 0.156) (Table 3; Figure 2). Detailed regression coefficients for all covariates are presented in Supplementary Table S2.

To examine the role of covariates, we conducted additional regression analyses predicting anxiety, depression, and marital adjustment (Hayes, 2022). Several covariates emerged as significant predictors. Higher education level was significantly associated with higher anxiety (*b* = 0.432, *p* = 0.019) but also with better marital adjustment (*b* = 3.282, *p* = 0.015). Higher monthly income was significantly associated with lower depression (*b* = −0.528, *p* = 0.002). Decreased sexual frequency after treatment was significantly associated with poorer marital adjustment (*b* = −7.818, *p* = 0.003). Additionally, living in Shenzhen (vs. other cities) was significantly associated with better marital adjustment (*b* = 3.251, *p* = 0.023).

Importantly, the inclusion of the 15 covariates did not substantially alter the main mediation effects. The serial indirect effect (resilience → anxiety → depression → marital adjustment) remained significant and similar in magnitude between the crude model (*b* = 0.235) and the adjusted model (*b* = 0.219), with overlapping confidence intervals. A full summary of all covariate effects is provided in Supplementary Table S2.

## 4 Discussion

Among Chinese patients with breast cancer, the present findings indicate that higher psychological resilience is positively associated with marital adjustment, not only directly but also indirectly through anxiety and depression. After adjustment for 15 sociodemographic and clinical covariates, the combined indirect effect accounted for 53.65% of the total association, a proportion that points to the sizable explanatory role of emotional mediators. Within the three indirect pathways specified in the serial mediation model, the pathway operating through anxiety alone (resilience → anxiety → marital adjustment) did not reach significance (*b* = 0.142, 95% CI [−0.141, 0.448]), which suggests that anxiety, on its own, may not connect resilience to marital outcomes unless depression is also involved. In contrast, the path via depression alone (resilience → depression → marital adjustment) was significant (*b* = 0.153, 95% CI [0.047, 0.292]), indicating depression’s direct mediating role. The serial pathway (resilience → anxiety → depression → marital adjustment) was also significant and the strongest (*b* = 0.219, 95% CI [0.069, 0.403]), explaining 22.86% of the total effect. This pattern implies that anxiety acts as an initial “entry” point, amplifying depression’s negative impact on marital dynamics, while resilience mitigates this cascade.

This pathway’s mechanism aligns with Stress-Coping Theory (SCT). According to SCT, resilient individuals view cancer diagnosis as a manageable challenge. This appraisal curbs initial anxiety escalation and blocks its progression to depressive symptoms. As a result, it improves patients’ subjective perception of marital adjustment. The serial mediation effect exceeds that of individual pathways. This positions anxiety as a key “entry” mediator, which is associated with greater depressive symptoms. Overall, these findings support the Vulnerability-Stress-Adaptation (VSA) model. They also highlight resilience’s pivotal role in disrupting cascading emotional distress.

The serial mediation model proposed in this study builds upon and extends previous research by addressing several gaps (Lai et al., 2020; Li et al., 2020). For instance, Brandão et al. (2017) reported that depression accounted for 15% of the variance in marital adjustment, whereas our model explains a larger proportion (approximately 25%), potentially due to the incorporation of a sequential pathway from anxiety to depression. This finding is consistent with prior literature on comorbidity trajectories in emotional disorders (El Kherchi et al., 2021; Zou et al., 2024). Similarly, Kong et al. (2024) and Li et al. (2015) confirmed resilience’s alleviation of anxiety or depression but overlooked chained effects. Recent Studies like Mohlin et al. (2021) and Fortin et al. (2021) highlight resilience’s protection of mental health but did not explore its relationship with marital functioning.

By focusing on a Chinese breast cancer population, this study also contributes cultural context to the literature, including findings related to sexual dysfunction (61%) that align with previous reports (Rodrigues-Machado et al., 2025; Yan et al., 2020). Differing from studies that focus on genetic determinants of psychological distress (Bayer et al., 2022), our work emphasizes modifiable psychosocial factors and includes a comprehensive covariate adjustment strategy involving 15 variables to reduce potential confounding. Although the cross-sectional design limits causal inference compared to longitudinal studies (Weber et al., 2023; Kalin, 2020), the model’s robustness was supported by bootstrap methods. Therefore, this study complements existing frameworks on relational stability (Dorval et al., 1999; Coyne, 2010) by incorporating serial emotional pathways, offering a more nuanced understanding of how resilience may influence marital adjustment in cancer contexts.

### 4.1 Clinical implications

These results advance psycho-oncology theory by affirming resilience’s enhancement of marital adaptation through emotional serial pathways. Clinically, our findings suggest several specific recommendations for intervention. First, interventions should prioritize the early reduction of anxiety, as it serves as the critical entry point that amplifies subsequent depressive symptoms and impairs marital adjustment. Cognitive reframing and uncertainty management techniques delivered shortly after diagnosis may effectively interrupt this cascade. This mechanism can guide nursing practices, integrating resilience assessments into routine screening, particularly for younger patients or those undergoing mastectomy (high-risk groups), to improve survival outcomes and reduce healthcare burdens (Ding et al., 2025). Furthermore, resilience-building interventions should target the five core domains of the CD-RISC-10: flexibility, self-efficacy, emotion regulation, optimism, and attention focus. For example, mindfulness training and problem-solving skills can enhance emotion regulation and flexibility, while benefit-finding exercises may strengthen optimism and self-efficacy (Loprinzi et al., 2011). Couple-based dyadic (Li et al., 2023; Tunç et al., 2023) and intelligent interventions should be implemented, such as mobile health (mHealth)-based psychological resilience interventions, to improve patients’ psychological resilience and reduce anxiety and depression (Zhang et al., 2025). These interventions may yield the greatest benefit when delivered during the active treatment phase (within the first year post-diagnosis), when anxiety levels are typically highest. Finally, these findings support the integration of resilience and emotional distress screening into stepped-care models, where patients with low resilience and elevated anxiety first receive low-intensity resilience training, and those with persistent difficulties are stepped up to more intensive couple-based interventions (Badr and Krebs, 2013). Future research should employ longitudinal designs to trace mediation dynamics and optimize phased interventions.

### 4.2 Limitations

Several limitations warrant attention. First, the cross-sectional design precludes causal inferences and prevents us from confirming the temporal sequence of anxiety and depression in the proposed mediation pathway. Although this sequence is supported by theoretical models and prior longitudinal research, alternative explanations such as reverse causality or unmeasured confounding cannot be ruled out (Chen et al., 2025). Second, the sample was relatively homogeneous, with the majority of participants being Han Chinese and diagnosed within 1 year, limiting generalizability to more diverse populations and patients with advanced disease. Third, several clinically important variables, including cancer stage, chemotherapy/radiotherapy/endocrine therapy status, and menopausal status, were not collected, which may introduce residual confounding. Fourth, this study only collected data from patients and did not include their partners. This individual-level approach limits our understanding of dyadic processes and mutual influences within couples. Future research should adopt a dyadic design by collecting data from both patients and their partners and applying the Actor-Partner Interdependence Model (APIM) to examine bidirectional effects. Future studies employing longitudinal designs, objective clinical data from medical records, and more heterogeneous samples are needed to validate and extend these findings.

## 5 Conclusion

This study reveals a novel mechanism by which resilience bolsters marital functioning among breast cancer patients by disrupting the progression of anxiety and depression. The findings illustrate the enduring nature of this sequence of impacts, even after considering various confounding variables, emphasizing the significance of the timing of emotional manifestations in couples’ adjustment to the challenges posed by the illness. These findings establish a link between individual coping theories and relational stress models, suggesting specific areas for targeted psychosocial interventions implemented sequentially. Strengthening resilience during pivotal junctures may yield dual benefits by improving psychological well-being and preserving intimate relationships, the latter of which plays a pivotal role in influencing survival outcomes.

## Data Availability

The datasets generated and analyzed during the current study are not publicly available due to institutional policies and participant privacy regulations, but are available from the corresponding author on reasonable request.
